# A methodology for supplier selection under the curse of dimensionality problem based on fuzzy quality function deployment and interval data envelopment analysis

**DOI:** 10.1371/journal.pone.0253917

**Published:** 2021-07-14

**Authors:** Xi Bao, Fenfen Li

**Affiliations:** 1 School of Business, Central South University, Changsha, P. R. China; 2 School of Public Administration, Hunan University of Finance and Economics, Changsha, P. R. China; 3 School of Public Administration, Xiangtan University, Xiangtan, China; University of Defence in Belgrade, SERBIA

## Abstract

Supplier selection is an important decision-making problem, which involves many quantitative and qualitative factors incorporating vagueness and imprecision. This study proposes a novel fuzzy multi-criteria decision-making framework for supplier selection, which integrates quality function deployment (QFD) and interval data envelopment analysis (DEA). The proposed methodology allows for considering the relationships among the product features and supplier evaluation criteria (SEs) and the impacts of inner dependence among SEs by constructing a house of quality (HOQ). Considering that the number of supplier evaluation indicators is greater than the number of suppliers in some cases, the curse of dimensionality problem usually exists. To solve this problem, we combine the HOQ, interval DEA models, and forward-stepwise selection approach to screen supplier evaluation indicators and select the best supplier(s). Through the two-stage supplier selection method, we can achieve the double screening of indicators and determine the final supplier(s). Finally, the application of the proposed framework is demonstrated through a numerical example and a sensitivity analysis is also carried out to verify the stability of the proposed methodology. This study focuses on supplier selection based on the combination of fuzzy QFD and interval DEA, and also provide a new two-phase methodology for DEA indicator screening.

## Introduction

Supplier selection decisions are viewed as critical problems in developing a strategically competitive position in the supply chain [1]. Having identified the necessity to well manage the supplier selection process, companies must seek a systematic and reasonable approach to avoid the consequences of poor decisions regarding supplier selection. In the process of selecting suppliers, companies not only pay attention to the direct economic benefits brought by the suppliers at the commercial level, but also pay attention to the indirect economic benefits brought by the suppliers’ sustainable development. Efficient supply chain management should not only consider the way that the products are produced but also take care of social, environmental and economic issues. To improve the competitiveness in the supply chain, a higher level of integration of suppliers and customers is required [2].

In the process of supplier selection, the ultimate aim of companies is to select suppliers that ensure a certain quality standard in terms of the characteristics of the purchased products or services [3]. To achieve this aim, we should consider the relationships between product features and supplier evaluation criteria (SEs) at the same time. Specifically, the relationships among SEs are considered to avoid the unrealistic independence assumption. As part of the quality function deployment (QFD) method, house of quality (HOQ) not only considers the relationships between product features and SEs but also the inner dependence of SEs. Consequently, constructing an HOQ is essential to determine how well each supplier characteristic succeeds in meeting the customer requirements (CRs) for products. According to the relative importance weightings of CRs for products, we can also compute and obtain those of SEs.

Data envelopment analysis (DEA) is a non-parametric multi-criteria decision-making technique based on linear programming, which is first proposed by [4]. DEA is used for comparing a set of homogenous decision-making units (DMUs) by evaluating their relative efficiency [1]. The original DEA models assume that inputs and outputs are measured by crisp values. However, in the real world, many inputs or outputs are variables with imprecise data [5]. Cooper et al. [6] first deal directly with imprecise data in DEA. In the same work, imprecise DEA is extended to AR-IDEA (imprecise DEA with assurance region), which includes the assurance region approach. Despotis and Smirlis [7] develop an alternative approach for dealing with imprecise data. Interval data, as one of the forms of imprecise data, are widely used to describe fuzzy conditions. Different from the imprecise DEA model proposed by [6], Despotis and Smirlis [7] transform the variables on the basis of the original dataset without applying any scale transformations on the data, which can estimate upper and lower bounds of the efficiency scores of DMUs and classify and further discriminate the DMUs in terms of the variability of their efficiency scores.

In the supplier evaluation process, many quantitative and qualitative factors are incorporated, and the data of these factors are usually full of vagueness and imprecision. In order to solve this problem, we select interval DEA models to evaluate candidate suppliers and select the best supplier(s). The inputs and outputs fed into the interval DEA models consist of the SEs involved in an HOQ. The relative importance weightings of SEs derived by the HOQ are incorporated in the interval DEA models as constraints. Jafarzadeh et al. [8] propose a three-phase methodology for project portfolio selection based on fuzzy QFD and DEA. They use a triangular fuzzy function to transform fuzzy numbers into crisp values, and then use the original DEA model to evaluate the maximal portfolio. Different from their paper, this study focuses on the combination of fuzzy QFD and interval DEA and always uses interval numbers for calculation and evaluation, which avoids the distortion of the conversion of interval numbers to precise numbers. Besides, this study also adds to indicator screening literature by introducing a two-phase methodology. In many cases, the dimensions of SEs are greater than the number of candidate suppliers. As DEA algorithm is sensitive to the number of input and output criteria in the model, the curse of dimensionality problem usually exists. That is, too many input and output indicators can weaken the discrimination power of evaluated DMUs. To avoid the curse of the dimensionality problem, we propose a forward-stepwise selection approach to identify these supplier evaluation indicators, which meet the core CRs for products.

The rest of this paper is organized as follows. Section 2 provides the basic background of this study (including supplier selection, interval data, and fuzzy QFD). Section 3 presents the proposed two-phase model framework. Section 4 provides an example to illustrate how the proposed methodology works and presents a sensitivity analysis of our proposed methodology. Section 5 makes a conclusion and provides directions for future research.

## Background

### Supplier selection

Supplier selection is the process in which companies identify, screen, evaluate, analyze, and contract with suppliers [9]. As suppliers have a substantial effect on the financial and supply chain performance of companies, finding suitable suppliers with high-quality services is viewed as a key task of companies [10]. More than 63% of supplier selection studies are found in multi-criteria environment [11].

In fact, supplier selection is a complicated issue and some researchers discuss this issue in different fields. Luthra et al. [12] propose a framework for evaluating the sustainable supplier selection and discuss a real-world example of an automobile company in India to demonstrate the proposed framework applicability. Alikhani et al. [13] consider sustainability and risk simultaneously, and further help a well-known Iranian supermarket chain, Shahrvand Goods & Servicing Company, to select reliable and sustainable suppliers. Yazdani et al. [14] apply an integrated decision-making model to orthopedic supplier selection of a Spanish hospital. Kannan et al. [15] propose a new framework for sustainable circular supplier selection, which is used to evaluate six suppliers in the wire-and-cable industry in Iran. Chen et al. [16] propose a novel rough-fuzzy approach for sustainable supplier selection and show the method’s feasibility with vehicle transmission supplier selection. Stević et al. [17] first study sustainable supplier selection in private healthcare industry. Besides, Stević et al. [18] perform the supplier selection in the construction company.

In recent years, many researchers have incorporated several approaches and new analyses into supplier selection. Chai et al. [19] emphasize the uncertainty environment in supplier selection, including stochastic information, gray numbers, and fuzzy variables, and its diversified family (e.g., triangular, trapezoidal, intuitionistic, and internal valued fuzzy variables). Loss aversion, together with risk aversion, has been applied for resolving supplier selection problems, such as in [20]. Dobos and Vörösmarty [21] adopt a DEA method considering environmental protection and sustainability in selecting green suppliers. Dey et al. [22] emphasize organizational and human factors in strategic supplier selection. Moreover, considerable research considers group and negotiation processes in supplier selection [23–25].

Considering the complexity of supplier selection, many decision-making techniques are applied. Existing literature about decision-making techniques used for supplier selection is mainly divided into three categories: multi-attribute decision-making techniques, mathematical programming techniques, and data mining and artificial intelligence (AI) techniques [26]. The first category involves Analytic Hierarchy Process (AHP) and Analytic Network Process (ANP), which have been integrated with other methods. For example, Lu et al. [27] use AHP and fuzzy logic in their assessment of environmentally aware suppliers. Tuzkaya et al. [28] model the environmental performance evaluation of suppliers in fuzzy ANP and fuzzy preference ranking organization method for the enrichment evaluation methodology. The second category contains classical and mixed programming techniques, among which linear programming is widely used for supplier selection [23]. Ho et al. [29] claim that DEA is the most general linear programming technique for the supplier selection problem. Chen et al. [30] develop a heuristic method that integrates mixed integer linear programming. Kuo and Lin [31] present a supplier selection method by using ANP and DEA. The third category is a new trend of supplier selection methods. To meet the needs of big data analysis, many new approaches have been reported and incorporated toolkits of data mining. Data mining and AI techniques focus on classification and clustering. Many traditional data mining approaches are used for supplier selection, such as neural networks, Bayesian networks, decision tree, and K-Means clustering [32–35].

To deal with numerous and conflicting criteria to rank and select the best suppliers of value chain framework, many researchers have recently combined QFD and other multi-criteria decision-making methods to assist supplier selection. The QFD and data mining methods are used by [36] for selecting and ranking suppliers. Bevilacqua et al. [37] use fuzzy numbers to QFD for the supplier selection problem. Gencer and Gürpinar [38] also utilize fuzzy QFD for the selection process of an Internet service provider.

### Interval data

Interval analysis is a widely used decision-making tool in situations where the data are unknown exactly but are known to lie within bounded intervals [39]. Let *a* = [*a*^*L*^, *a*^*U*^] = {*x*│*a*^*L*^ ≤ *x* ≤ *a*^*U*^} and *b* = [*b*^*L*^, *b*^*U*^] = {*x*│*b*^*L*^ ≤ *x* ≤ *b*^*U*^} be the two interval fuzzy numbers. We can find that if *a*^*L*^ = *a*^*U*^, then *a* becomes a real number. The algebraic operations of these two interval fuzzy numbers are defined as follows:

1. Addition and subtraction of two interval fuzzy numbers:
$$a\pm b=\left[a^{L}\pm b^{L},\;a^{U}\pm b^{U}\right]$$ (1)
2. Multiplication of two interval fuzzy numbers:
$$a\times b=\left[a^{L}\times b^{L},\;a^{U}\times b^{U}\right]$$ (2)
3. Division of two interval fuzzy numbers:
$$a÷b=\left[a^{L}÷b^{U},\;a^{U}÷b^{L}\right]$$ (3)
4. Multiplication of interval fuzzy numbers by a non-negative constant *r*:
$$r\times a=\left[r\times a^{L},\;r\times a^{U}\right].$$ (4)

### Fuzzy QFD

**Fig 1:**
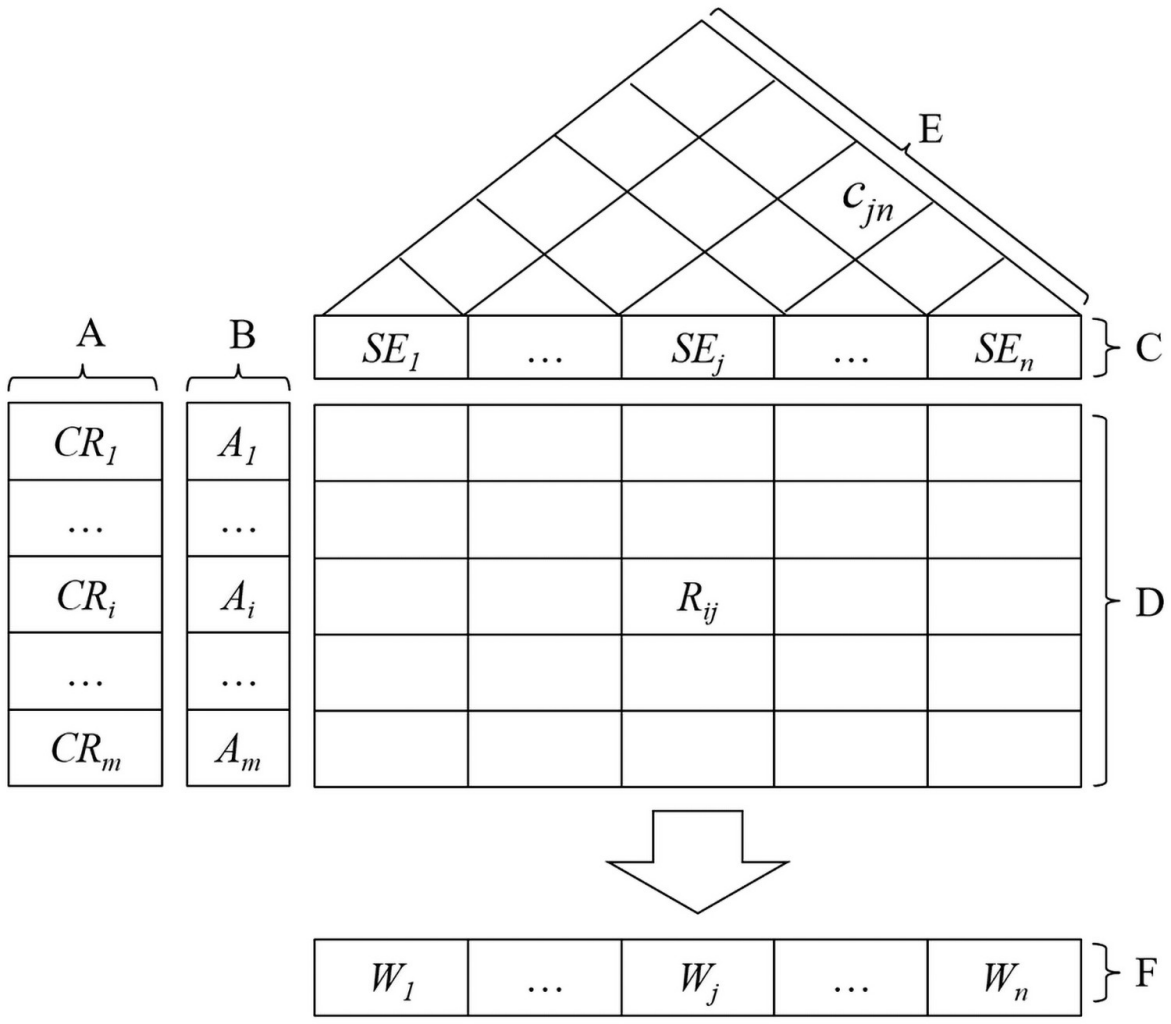
HOQ.

QFD is a comprehensive and widely known quality management tool, which originated in the 1960s to translate CRs into the characteristics of new services and products [40]. In the QFD approach, CRs for products or services are transformed into detailed qualitative and quantitative requirements, which help engineers identify the features and characteristics of a product or service. In addition to being used to establish the connection between customer needs and product functions, QFD has also been applied to many other fields, including project portfolio selection and supplier selection [41, 42]. In general, QFD is suitable for those situations where objectives are prioritized based on the importance of requirements. For supplier selection, to establish the relationships between CRs and SEs, the QFD approach is applied on the basis of a semantic visualization called “HOQ” (Fig 1), which facilitates transforming CRs into SEs.

According to Fig 1, HOQ is mainly composed of six parts [43]. The QFD process begins with experts (or other decision makers [DMs]) identifying the CRs (block A) and further determining the relative importance of each CR based on customer surveys (block B). The experts then transform CRs into several SEs based on their judgments and experiences (block C). In the next step, the experts judge which CR impacts an SE and to what extent (block D). The correlation between the SEs is also identified by the experts (block E). The importance weighting of each supplier evaluation criterion is calculated (block F) using the data from blocks A to E, which is the aim of the HOQ process. These weightings are then used in the process of supplier selection by informing which supplier(s) are appropriate to ensure that the CRs with relevant high importance are best satisfied.

Traditionally, all importance scores and weightings in an HOQ are set as crisp values [44], but decision making in the real world is always tied to vagueness and imprecision. In practical circumstances, limiting the judgments to crisp values has always been regarded as one of the difficult problems. Therefore, the combination of interval analysis and QFD can greatly solve this problem in practical decision-making circumstances [41], allowing the incorporation of unquantifiable and incomplete information [45]. Fuzzy QFD has been introduced by some researchers incorporating interval fuzzy numbers and algebraic operations [46–48]. In these studies, interval fuzzy numbers are used to quantitatively describe linguistic judgments of DMs. We use this approach (interval fuzzy numbers and algebraic operations) in our study, which will be explained later in the proposed method [Section 3]).

### DEA

Data envelopment analysis (DEA) is one of the most frequently used multiple-criteria decision-making methods in supplier selection problems [49]. It helps to choose optimal weight of each DMU which affects the results of the supplier selection process. Narasimhan et al. [50] propose a methodology with the help of DEA for evaluation to identify supplier clusters. Talluri et al. [51] propose a novel method which is anchored in cross efficiency analysis in DEA that allows for evaluating the efficiency of a supplier with respect to the optimal weights of its peers. Kumar et al. [52] develop a green DEA (GDEA) method which incorporates heterogeneous suppliers and regional emission compliance standards and laws. Dobos and Vörösmarty [53] apply DEA to evaluate suppliers based on management criteria and green criteria.

In addition to using the DEA method separately, many researchers try to combine DEA with other decision-making methods or theories in the supplier selection. Kuo and Lin [31] present a supplier selection method by using analysis network process as well as DEA. Mahdiloo et al. [54] integrate multiple objective linear programming with DEA to measure eco-efficiency properly. Karsak and Dursun [55] present a novel fuzzy multi-criteria group decision making framework that integrates QFD and DEA for supplier selection. Jafarzadeh et al. [8] propose a three-phase methodology for project portfolio selection based on fuzzy QFD and DEA. In this study, we integrate fuzzy QFD, DEA and interval analysis for supplier selection and help solve the curse of dimensionality problem in DEA.

The original DEA models assume that inputs and outputs are measured by crisp values. However, in the real world, many inputs or outputs are variables with imprecise data [5]. Cooper et al. [6] first deal directly with imprecise data in DEA. In the same work, imprecise DEA is extended to AR-IDEA (imprecise DEA with assurance region), which includes the assurance region approach. Despotis and Smirlis [7] develop an alternative approach for dealing with imprecise data. Interval data, as one of the forms of imprecise data, are widely used to describe fuzzy conditions. Different from the imprecise DEA model proposed by [6], Despotis and Smirlis [7] transform the variables on the basis of the original dataset without applying any scale transformations on the data, which can estimate upper and lower bounds of the efficiency scores of DMUs and classify and further discriminate the DMUs in terms of the variability of their efficiency scores.

To estimate upper and lower bounds of the efficiency scores of the DMUs and further discriminate the DMUs in terms of the variability of their efficiency scores, we introduce the interval DEA model proposed in [7] to measure the efficiency of the candidate suppliers. Assume that *n* DMUs exist, and each DMU uses *m* inputs to produce *s* outputs. For DMU *j* (*j* = 1, ⋯, *n*), denote *y*_*rj*_ as the rth output (*r* = 1, ⋯, *s*) and *x*_*ij*_ as the ith input (*i* = 1, ⋯, *m*). Different from the original DEA model, the values of input–output variables are not crisp but are known to lie within bounded intervals, that is, $x_{ij}\in \left[x_{ij}^{L},\;x_{ij}^{U}\right]$ and $y_{rj}\in \left[y_{rj}^{L},\;y_{rj}^{U}\right]$. The upper and lower bounds of the intervals are given as constants and assumed strictly positive. In such a setting, the following model provides an upper bound of interval efficiency for DMU *j*_0_:

$$\begin{array}{lll} max & h_{j_{0}}^{U}=\sum_{r=1}^{s}u_{r}y_{rj_{0}}^{U} & \\ s.t. & \sum_{i=1}^{m}v_{i}x_{ij_{0}}^{L}=1, & \\ & \sum_{r=1}^{s}u_{r}y_{rj_{0}}^{U}-\sum_{i=1}^{m}v_{i}x_{ij_{0}}^{L}\leq 0, & \\ & \sum_{r=1}^{s}u_{r}y_{rj}^{L}-\sum_{i=1}^{m}v_{i}x_{ij}^{U}\leq 0, & j=1,\;\cdots ,\;n;j\neq j_{0}\\ & u_{r},\;v_{i}\geq \varepsilon \;\;\forall r,\;i. & \end{array}$$ (5)

where variables *u*_*r*_ and *v*_*i*_ represent weights for outputs and inputs, respectively. The model below provides a lower bound of the efficiency scores for DMU *j*_0_:

$$\begin{array}{lll} max & h_{j_{0}}^{L}=\sum_{r=1}^{s}u_{r}y_{rj_{0}}^{L} & \\ s.t. & \sum_{i=1}^{m}v_{i}x_{ij_{0}}^{U}=1, & \\ & \sum_{r=1}^{s}u_{r}y_{rj_{0}}^{L}-\sum_{i=1}^{m}v_{i}x_{ij_{0}}^{U}\leq 0, & \\ & \sum_{r=1}^{s}u_{r}y_{rj}^{U}-\sum_{i=1}^{m}v_{i}x_{ij}^{L}\leq 0, & j=1,\;\cdots ,\;n;j\neq j_{0}\\ & u_{r},\;v_{i}\geq \varepsilon \;\;\forall r,\;i. & \end{array}$$ (6)

Through Models (5) and (6), a bounded interval $\left[h_{j}^{L},\;h_{j}^{U}\right]$ of each DMU can be obtained, indicating its possible efficiency in the worst and the best cases. On the basis of the efficiency intervals, the evaluated DMU can be classified into three subsets as follows:

$$E^{++}=\{j\in J|h_{j}^{L}=1\},$$ (7)

$$E^{+}=\left\{j\in J|h_{j}^{L}<1\;and\;h_{j}^{U}=1\right\},$$ (8)

$$E^{-}=\{j\in J|h_{j}^{U}<1\},$$ (9)

where *j* stands for the index set {1, ⋯, *n*} of the DMUs. The classification means that set *E*^++^ consists of the units that are efficient in any case, set *E*^+^ comprises units that are efficient in a maximal sense, and set *E*^−^ consists of the definitely inefficient units. Despotis and Smirlis [7] point out that the range of interval efficiency scores can be used to rank further the DMUs in set *E*^+^. That is, the smaller the difference $h_{j}^{U}-h_{j}^{L}$, the higher the ranking.

## Methodology

**Fig 2:**
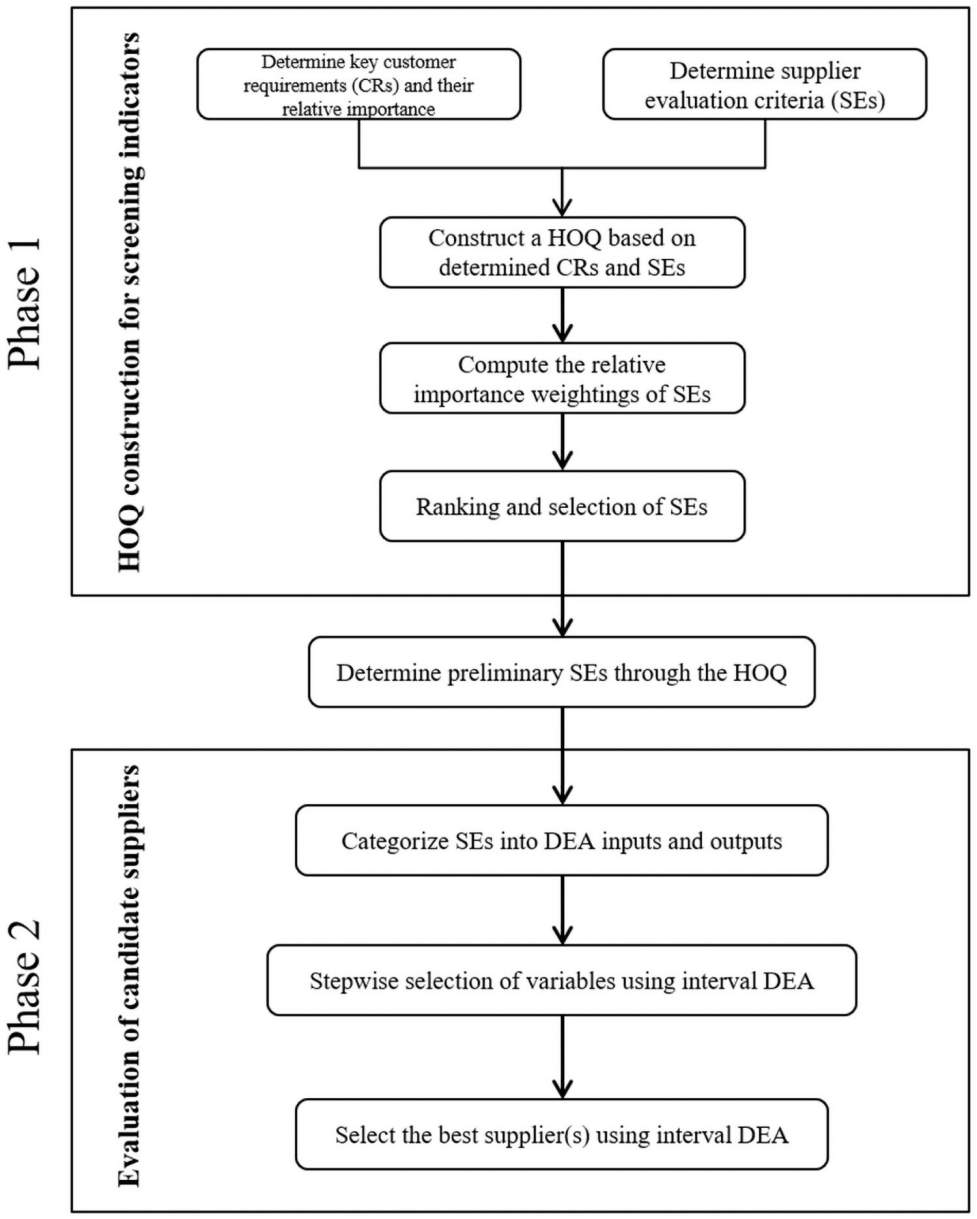
Presentation of the proposed integrated supplier selection methodology.

In this study, we propose a new decision-making framework which combines fuzzy QFD with interval DEA (Fig 2). Along the lines of the two-phase supplier selection method in [8], the proposed methodology includes two phases: (i) HOQ construction for screening indicators and (ii) evaluation of candidate suppliers. In the first phase, we determine the CRs and the initial SEs. On the basis of CRs and SEs, we construct an HOQ and compute the importance weightings of SEs. All initial SEs are ranked according to the importance weightings, and unimportant indicators are eliminated. In the second phase, we divide the screened SEs into inputs and outputs and use interval DEA to evaluate candidate suppliers. In this phase, if a certain indicator has a small impact on the efficiency values of DMUs, then we remove it and choose another indicator that has a smaller importance weighting in the first phase. Each phase is composed of several steps, which are described in the following.

### Phase 1. HOQ construction for screening indicators

#### Step 1a. Determine key CRs and their relative importance

Step 1 starts with choosing the key CRs. We first construct a decision maker committee of *z* experts (*x* = 1, 2, …, *z*). Then, we identify CRs. In the case of a certain product, the key characteristics that the product being purchased must possess should be identified to meet the CRs, and these characteristics are denoted as CRs. Once CRs are determined, the DMs judge their importance by using interval numbers. In our proposed method, we use relatively simple but widely-used linguistic terms, such as very high (VH), high (H), medium (M), low (L), and very low (VL) [3, 42]. Table 1 shows the interval fuzzy membership function used in this study. This function is chosen mainly for the convenience of calculation. In practical applications, the interval membership function has been one of the most frequently used forms of fuzzy numbers [56]. In this study, the upper and lower bounds of the intervals are given as constants and are assumed strictly positive.

**Table 1:** Linguistic terms used in this study.

Linguistic term	Interval value
Very high	[0.8, 1]
High	[0.6, 0.8]
Medium	[0.4, 0.6]
Low	[0.2, 0.4]
Very low	[0, 0.2]

After the linguistic judgments are converted into interval numbers by using the interval fuzzy membership function outlined above, they are aggregated using the average operator. For the sake of simplicity, here we aggregate interval numbers using simple arithmetic average, so as to avoid complicated average operators that affect the comprehensibility of this methodology.

Assume $A_{ix}\;=\;(a_{ix}^{L},\;a_{ix}^{U})$ as the interval fuzzy number of CRs that shows the opinion of the *x*^*th*^ expert (*x* = 1, 2, ⋯, *z*) about the *i*^*th*^ requirement (*i* = 1, 2, ⋯, *m*). The aggregated judgments of the experts regarding the *i*^*th*^ CR, represented by ${\tilde{A}}_{i}$, are calculated by:

$$\begin{array}{lll} {\tilde{A}}_{i}=\frac{1}{z}\left(\sum_{x=1}^{z}A_{ix}\right) & i=1,\;\cdots ,\;m & x=1,\;\cdots ,\;z. \end{array}$$ (10)

#### Step 1b. Determine SEs

After reviewing the supply management literature, experts are presented with various SEs. The factors should be integrated into SEs as comprehensively as possible without duplication. These factors serve as the candidate SEs later on in the process when the HOQ structure is constructed for the fuzzy QFD analysis.

#### Step 1c. Construct an HOQ on the basis of the determined CRs and SEs

After identifying the SEs and CRs for products, we construct the central relationship matrix (CRs–SEs) by using expertise knowledge, which displays the degree of relationship between each CR and the corresponding supplier evaluation criterion. Each expert is asked to express an opinion on the impact of each “SE” on each “CR.” Such an impact is recorded as a linguistic variable (e.g., VH, H, M, L, and VL). Here, interval numbers are also used to quantify the impacts and, as in the previous step, the interval data obtained from each expert are aggregated by the means proposed above. Let $R_{ijx}\;=\;(r_{ix}^{L},\;r_{ix}^{U})$ be the linguistic judgment of the relationship between the *i*^*th*^ customer requirement (*i* = 1, 2, ⋯, *m*) and *j*^*th*^ supplier evaluation criterion (*j* = 1, 2, ⋯, *n*), made by the *x*^*th*^ expert (*x* = 1, 2, ⋯, *z*). The aggregated judgments of the experts regarding the relationship between the *i*^*th*^ customer requirement and the *j*^*th*^ supplier evaluation criterion, represented by *R*_*ij*_, are calculated by:

$$\begin{array}{llll} R_{ij}=\frac{1}{z}\left(\sum_{x=1}^{z}R_{ijx}\right) & i=1,\;\cdots ,\;m & j=1,\;2,\;\cdots ,\;n & x=1,\;\cdots ,\;z. \end{array}$$ (11)

Therefore, the relationship matrix, which is the essence of the HOQ, is constructed. Correlations *c*_*kjx*_ between SEs themselves (“roof” matrix) are determined by the opinions of experts, which are also described using interval numbers. In this case, we can use the same method, and the aggregated judgments of the experts regarding the degree of dependence among SEs, represented by *c*_*kj*_, are calculated by:

$$\begin{array}{llll} c_{kj}=\frac{1}{z}\left(\sum_{x=1}^{z}c_{kjx}\right) & j=1,\;\cdots ,\;n & k=1,\;\cdots ,\;n & x=1,\;\cdots ,\;z. \end{array}$$ (12)

To eliminate differences between different judgments, the normalized central relationship matrix values are obtained by the general model as proposed by [57]:

$$\begin{array}{llll} {\tilde{R}}_{ij}=\frac{\sum_{k=1}^{n}R_{ik}c_{kj}}{\sum_{j=1}^{n}\sum_{k=1}^{n}R_{ik}c_{kj}} & i=1,\;\cdots ,\;m & j=1,\;\cdots ,\;n & k=1,\;\cdots ,\;n, \end{array}$$ (13)

where ${\tilde{R}}_{ij}$ represents the normalized relationship between *i*^*th*^ customer requirement and *j*^*th*^ supplier evaluation criterion, *R*_*ik*_ represents the quantified relationship between *i*^*th*^ customer requirement and kth supplier evaluation criterion, and *c*_*kj*_ represents the quantified relationship between kth supplier evaluation criterion and *j*^*th*^ supplier evaluation criterion.

#### Step 1d. Compute the relative importance weightings of SEs

The weighted mean of the *j*^*th*^ supplier evaluation criterion, ${\tilde{W}}_{j}^{*}\;=\;(w_{j*},\;w_{j}^{*})$, is calculated as:

$$\begin{array}{ll} {\tilde{W}}_{j}^{*}=\sum_{i=1}^{m}{\tilde{R}}_{ij}\times {\tilde{A}}_{i} & j=1,\;\cdots ,\;n. \end{array}$$ (14)

Next, the absolute importance weighting of the *j*^*th*^ criterion, $W_{j}^{*}$, is calculated as:

$$\begin{array}{ll} W_{j}^{*}=\frac{w_{j*}+\;w_{j}^{*}}{2} & j=1,\;\cdots ,\;n. \end{array}$$ (15)

Then, the absolute weighting of the *j*^*th*^ criterion is converted to the relative importance weighting *WC*_*j*_ according to:

$$WC_{j}=\frac{W_{j}^{*}}{\sum_{j=1}^{n}W_{j}^{*}}.$$ (16)

#### Step 1e. Rank and select SEs

The SEs can be ranked according to their relative importance levels, and the factors that do not have significant impacts on CRs can be removed. Thus, if the number of SEs is too high, then some SEs that are found to be less important should be left out. The relative importance threshold can be set by the decision makers, such as 0.05. In this way, the decision makers can easily compress the total number of SEs initially and focus on more important SEs.

### Phase 2. Evaluation of candidate suppliers

The first phase identifies and prioritizes CRs and SEs. Now, these SEs can be used as a set of criteria to determine the best supplier for organizations. In Phase 2, interval DEA method is incorporated to evaluate the candidate suppliers.

#### Step 2a. Categorize SEs into DEA inputs and outputs

In this study, SEs need to be classified as DEA inputs and outputs. When the evaluated process represents a production process, the resources used are the inputs and the outcomes are the outputs [58]. From the perspective of SEs, those SEs related to planning or operation strategies are clearly resources while other SEs related to objectives or revenue are regarded as outcomes of the production process.

#### Step 2b. Stepwise selection of variables using interval DEA

The DEA algorithm is sensitive to the number of input and output criteria in the model. Excessive input or output variables can reduce the effectiveness of DEA ranking in practice, which is called the curse of dimensionality. Referring to (17), the total number of input and output variables should be smaller than the number of DMUs divided by 3 [59]. In this study, it means that the total number of SEs (inputs plus outputs) fed into the DEA algorithm should be less than one-third of the number of suppliers. Thus, if the number of evaluation criteria is too high, then some evaluation criteria that are found to be less important should be removed from the DEA algorithm.

$$I+O<\frac{N}{3},$$ (17)

where *N* represents the number of DMUs (i.e., suppliers), *I* represents the number of inputs, and *O* represents the number of outputs.

In the supplier selection problem, some supplier evaluation indicators are fuzzy or interval data. In this way, we introduce the interval DEA model proposed above to select appropriate variables. To screen the supplier evaluation indicators, we incorporate the relative importance weightings of SEs computed in Step 1d as the additional constraints (Eq 18) of the interval DEA models. The input and output data are then fed into the interval DEA procedure to calculate the efficiency of each DMU.

$$v\in V,\;u\in U,\;\left(V\neq \emptyset ,\;U\neq \emptyset \right),$$ (18)

where *V* and *U* stand for the set of magnitudes of relative importance weightings of inputs and outputs, respectively.

To remove the less important criteria that weaken the discrimination power of the DEA algorithm, we use the forward-stepwise procedure for the modeling of DEA variables. The forward-stepwise approach starts by considering one input and one output in the DEA model. At each step, one variable is added to the model, and then the changes in the number of efficient DMUs are analyzed. Theoretically, the method can continue until all input and output variables are added to the model. In this study, the stopping rule is related to the number of efficient DMUs. The forward-stepwise algorithm, which is based on the relative importance weightings of criteria, is shown as follows:

For sets of *i* = 1, ⋯, *m* inputs and *r* = 1, ⋯, *s* outputs, assume that each input and output have relative importance weighting *v*_*i*_ and *u*_*r*_, respectively.

Start: Run a single DEA analysis that includes a set of one input and one output. Note that the selected input has the highest relative importance weighting among *m* inputs, and the selected output has the highest relative importance weighting among *s* outputs. Record the number of efficient DMUs for this run (set Q*). Let *C* = {*I*_0_, *O*_0_}.

Step 1: Run a DEA analysis, adding input or output *H*_1_ with the highest relative importance weighting among the remaining (*m* + *s* − 2) variables. Record the number of efficient DMUs for this run (set *Q*_1_). If *Q*_1_ > Q*, then C* = {*I*_0_, *O*_0_}. Stop. Otherwise, let *C* = {*I*_0_, *O*_0_, *H*_1_} and proceed to the next step.

……

Step t: Run a DEA analysis, adding input or output *H*_*t*_ with the highest relative importance weighting among the remaining (*m* + *s* − *t* − 1) variables. Record the number of efficient DMUs for this run (set *Q*_*t*_). If *Q*_*t*_ > Q*, then C* = {*I*_0_, *O*_0_, *H*_1_, ⋯, *H*_*t*−1_}. Stop. Otherwise, let *C* = {*I*_0_, *O*_0_, *H*_1_, ⋯, *H*_*t*_} and proceed to the next step.

……

Step (*m* + *s* − 2): Run a DEA analysis, adding the last variable *H*_*m*+*s*−2_. Record the number of efficient DMUs for this run (set *Q*_*m*+*s*−2_). If *Q*_*m*+*s*−2_ > Q*, then C* = {*I*_0_, *O*_0_, *H*_1_, ⋯, *H*_*m*+*s*−3_}. Otherwise, let C* = {*I*_0_, *O*_0_, *H*_1_, ⋯, *H*_*m*+*s*−2_}. Stop.

Note that from Steps 1 to *m* + *s* − 2, the algorithm can be terminated in each step. After the algorithm ends, we obtain the final evaluation criterion set C*, which can be used for supplier evaluation and selection.

#### Step 2c. Select the best supplier(s) using interval DEA

In this step, interval DEA is used to evaluate and select the best supplier(s) on the basis of the input and output criteria C* identified in the previous step. The best suppliers satisfy CRs and SEs, which combine the advantages of QFD and DEA at the same time. By adding the relative importance degrees of SEs as constraints, efficient DMUs can be identified using the interval DEA model.

## Numerical example

In this study, a numerical example is used to demonstrate an application of the proposed methodology. In the following, we illustrate how the proposed methodology can help with the supplier selection. For the scope of this example, seven suppliers (i.e., DMUs) are considered.

### Phase 1. HOQ construction for screening indicators

#### Step 1a. Determine key CRs and their relative importance

Phase 1 begins with the formation of a team of expert DMs and selection of four experts as consultants. Once the DM team is formed, the next step is to determine the list of CRs for products. Here, we choose the CRs from the case study conducted in a private hospital in the Asian side of Istanbul [56]. Five fundamental characteristics (CRs) required of products purchased from medical suppliers are determined, as presented in Table 2.

**Table 2:** Determined CRs for the medical products.

Row	Meaning
CR_1_	Cost
CR_2_	Quality
CR_3_	Product conformity
CR_4_	Availability and customer support
CR_5_	Efficacy of corrective action

Each DM is required to evaluate the relevant importance of each CR in an interval manner. Each DM needs to independently evaluate the importance of each CR according to the linguistic judgment rule (i.e., VH, H, M, L, and VL) (Table 3).

**Table 3:** Linguistic evaluations of CRs by DM 1–4.

CR	DM1	DM2	DM3	DM4
CR_1_	VH	H	L	L
CR_2_	VH	VH	H	H
CR_3_	H	H	L	M
CR_4_	VH	H	H	L
CR_5_	H	VL	M	L

Then, the judgments of DMs are aggregated with the average operator (Table 4) by using the interval fuzzy membership function outlined in Table 1.

**Table 4:** Aggregation of the linguistic evaluations of CRs.

CR	DM1	DM2	DM3	DM4	$\tilde{A_{i}}\;=\;\frac{1}{z}\left(\sum_{x\;=\;1}^{z}A_{ix}\right)$
CR_1_	[0.8, 1]	[0.6, 0.8]	[0.2, 0.4]	[0.2, 0.4]	[0.45, 0.65]
CR_2_	[0.8, 1]	[0.8, 1]	[0.6, 0.8]	[0.6, 0.8]	[0.7, 0.9]
CR_3_	[0.6, 0.8]	[0.6, 0.8]	[0.2, 0.4]	[0.4, 0.6]	[0.45, 0.65]
CR_4_	[0.8, 1]	[0.6, 0.8]	[0.6, 0.8]	[0.2, 0.4]	[0.55, 0.75]
CR_5_	[0.6, 0.8]	[0, 0.2]	[0.4, 0.6]	[0.2, 0.4]	[0.3, 0.5]

#### Step 1b. Determine SEs

In this example, the SEs are also from [44]. Determining the most preferred supplier depends on some distinct features. These criteria relevant to supplier evaluation are identified in Table 5. Among these factors, product volume, supply variety, and reliability are directly related to products. Delivery and payment method are related to the final delivery process of products. The four other criteria reflect the production and operations of suppliers.

**Table 5:** Determined SEs for the medical products.

Row	Meaning
SE_1_	Product volume
SE_2_	Delivery
SE_3_	Payment method
SE_4_	Supply variety
SE_5_	Reliability
SE_6_	Experience in the sector
SE_7_	Earlier business relationship
SE_8_	Management
SE_9_	Geographical location

#### Step 1c. Construct an HOQ on the basis of determined CRs and SEs

**Fig 3:**
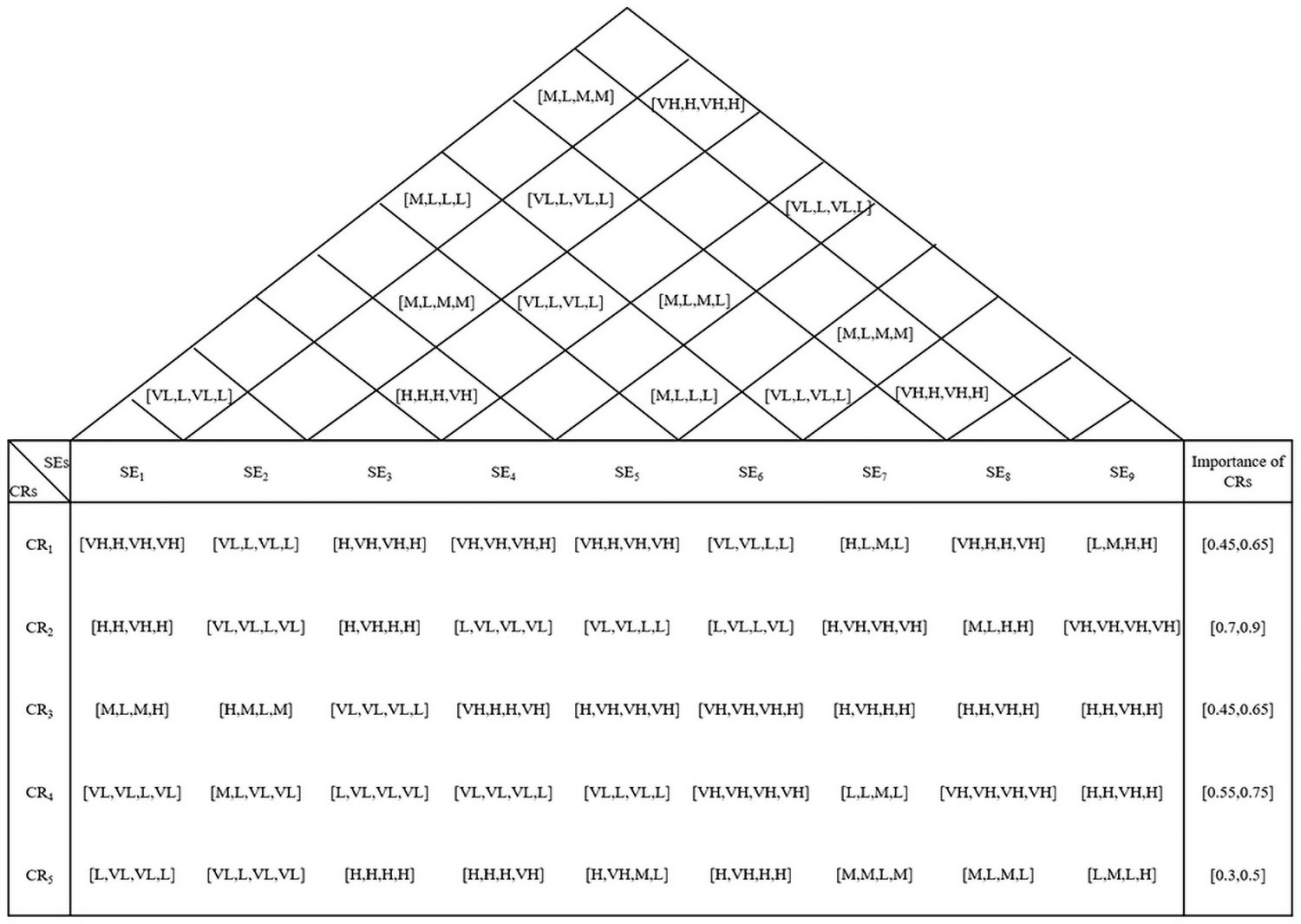
Basic HOQ for the medical supplier selection problem.

After the SEs and CRs are identified, a basic HOQ is constructed (Fig 3) using expert knowledge, which shows the degree of relationship between each CR and each SE and the degree of dependence of one SE on another SE employing linguistic variables defined in Table 1. In Fig 3, each element of HOQ represents the judgments of four experts. For example, (*VH*, *H*, *VH*, *VH*) means that the judgments of four experts are VH, H, VH, VH, respectively. The rightmost column in Fig 3 shows the importance of CRs which is calculated in step 1a (Table 4).

#### Step 1d. Compute the relative importance degrees of SEs

**Fig 4:**
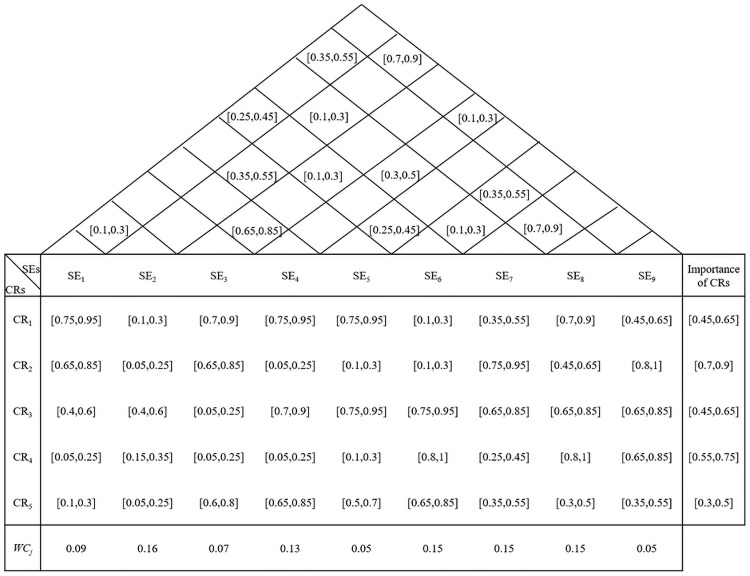
Final HOQ for the medical supplier selection problem.

The aggregated impact of each SE on each CR and aggregated degree of dependence of SEs are obtained by using Eqs (11) and (12), which are displayed in Fig 4. Based on the relationship between the central matrix and “roof” matrix illustrated in Fig 4, we can use Eq (13) to determine a normalized relationship between CRs and SEs, $\tilde{R_{ij}}$ and then use Eqs (14)–(16) to calculate the relative importance degree of each SE, *WC*_*j*_. They are calculated as 0.09, 0.16, 0.07, 0.13, 0.05, 0.15, 0.15, 0.15, and 0.05, which are shown in the last row of Fig 4. The supplier evaluation criterion SE_2_ (delivery) has the highest importance weighting (0.16). The results show that placing high emphasis on delivery when selecting suppliers can improve the customer satisfaction degree.

#### Step 1e. Rank and cut off SEs

In the previous step, we calculate the relative importance degrees on the SEs. The final weights and rankings of nine criteria are shown in Table 6. The results reveal that the relative importance degrees of these nine criteria are not far apart. Therefore, we choose to retain all the criteria and turn to the next phase.

**Table 6:** Final weights and rankings of SEs.

SE	Meaning	*WC*_*j*_	Rank
SE_2_	Delivery	0.16	1
SE_6_	Experience in the sector	0.15	2
SE_7_	Earlier business relationship	0.15	2
SE_8_	Management	0.15	2
SE_4_	Supply variety	0.13	5
SE_1_	Product volume	0.09	6
SE_3_	Payment method	0.07	7
SE_5_	Reliability	0.05	8
SE_9_	Geographical location	0.05	8

### Phase 2. Evaluation of candidate suppliers

The weights of SEs obtained in Phase 1 (Table 6) lays the foundation for the selection of the optimal supplier in Phase 2. In the following, we take our example through the three steps of Phase 2 to identify the best supplier.

#### Step 2a. Categorize SEs into DEA inputs and outputs

The data in Table 7 consist of linguistic judgments, which are converted into interval data, as shown in Table 8.

**Table 7:** Linguistic ratings of suppliers with respect to SEs.

	Sup 1	Sup 2	Sup 3	Sup 4	Sup 5	Sup 6	Sup 7
SE_1_	(VH,VH,VH,VH)	(H,H,M,VH)	(M,L,M,M)	(M,M,L,M)	(M,L,M,M)	(H,H,H,H)	(H,H,VH,VH)
SE_2_	(H,H,M,H)	(VH,H,H,VH)	(VH,VH,H,VH)	(H,VH,VH,VH)	(H,M,H,VH)	(M,M,H,H)	(H,M,M,H)
SE_3_	(VH,H,H,VH)	(H,VH,M,M)	(VH,H,H,H)	(H,VH,VH,H)	(VH,VH,H,VH)	(VH,H,VH,VH)	(H,VH,VH,VH)
SE_4_	(VH,VH,VH,VH)	(H,M,H,H)	(M,L,M,H)	(M,M,L,H)	(M,H,M,M)	(H,VH,H,H)	(M,H,H,H)
SE_5_	(H,VH,H,VH)	(H,H,VH,H)	(H,M,M,H)	(VH,M,H,VH)	(M,H,L,L)	(M,H,H,H)	(H,H,H,VH)
SE_6_	(VH,VH,VH,VH)	(H,VH,VH,H)	(H,H,H,H)	(VH,VH,H,VH)	(M,L,M,M)	(M,H,L,M)	(VH,VH,VH,VH)
SE_7_	(H,VH,H,H)	(H,H,H,VH)	(VH,H,VH,VH)	(VH,VH,H,VH)	(H,VH,H,H)	(VH,VH,H,VH)	(H,VH,H,VH)
SE_8_	(M,M,H,H)	(H,M,H,H)	(M,H,M,H)	(H,M,H,H)	(L,M,L,VL)	(M,H,H,M)	(H,H,VH,VH)
SE_9_	(L,M,M,VL)	(M,L,L,L)	(VH,H,H,VH)	(M,L,M,H)	(M,L,M,M)	(L,L,M,L)	(M,M,L,M)

**Table 8:** Interval ratings of suppliers with respect to SEs.

Sup	Outputs					Inputs			
	SE_1_	SE_2_	SE_3_	SE_4_	SE_5_	SE_6_	SE_7_	SE_8_	SE_9_
Sup 1	[0.8,1]	[0.55,0.75]	[0.7,0.9]	[0.8,1]	[0.7,0.9]	[0.8,1]	[0.65,0.85]	[0.5,0.7]	[0.25,0.45]
Sup 2	[0.6,0.8]	[0.7,0.9]	[0.55,0.75]	[0.55,0.75]	[0.65,0.85]	[0.7,0.9]	[0.65,0.85]	[0.55,0.75]	[0.25,0.45]
Sup 3	[0.35,0.55]	[0.75,0.95]	[0.65,0.85]	[0.4,0.6]	[0.5,0.7]	[0.35,0.55]	[0.75,0.95]	[0.5,0.7]	[0.7,0.9]
Sup 4	[0.35,0.55]	[0.75,0.95]	[0.7,0.9]	[0.4,0.6]	[0.65,0.85]	[0.75,0.95]	[0.75,0.95]	[0.55,0.75]	[0.4,0.6]
Sup 5	[0.35,0.55]	[0.6,0.8]	[0.75,0.95]	[0.45,0.65]	[0.35,0.55]	[0.35,0.55]	[0.65,0.85]	[0.2,0.4]	[0.35,0.55]
Sup 6	[0.6,0.8]	[0.5,0.7]	[0.75,0.95]	[0.65,0.85]	[0.55,0.75]	[0.4,0.6]	[0.75,0.95]	[0.5,0.7]	[0.25,0.45]
Sup 7	[0.7,0.9]	[0.5,0.7]	[0.75,0.95]	[0.55,0.75]	[0.65,0.85]	[0.8,1]	[0.7,0.9]	[0.7,0.9]	[0.35,0.55]

In Table 8, among the nine SEs listed in Table 6, we categorize five of them, namely, product volume (SE_1_), delivery (SE_2_), payment method (SE_3_), supply variety (SE_4_), and reliability (SE_5_) as outputs because they are primarily about product characteristics. We categorize the four other factors, namely, experience in the sector (SE_6_), earlier business relationship (SE_7_), management (SE_8_), and geographical location (SE_9_) as inputs because they are relevant to the business operations of suppliers toward producing products.

#### Step 2b. Stepwise selection of variables using interval DEA

Considering that the DEA algorithm is sensitive to the number of input and output criteria in the model, we perform the stepwise selection of variables by using interval DEA models (5) and (6). The relative importance degrees of SEs in Table 6 are incorporated as the additional constraints of the interval DEA models. According to the description in Section 3, the evaluated DMU can be classified in three subsets, namely, *E*^++^, *E*^+^, and *E*^−^. The classification means that set *E*^++^ consists of the units that are efficient in any case, set *E*^+^ comprises units that are efficient in a maximal sense, and set *E*^−^ consists of the definitely inefficient units. Despotis and Smirlis [7] point out that the range of interval efficiency scores can be used to rank further the DMUs in set *E*^+^.

That is, the smaller the difference $h_{j}^{U}-h_{j}^{L}$, the higher the ranking. Therefore, we can rank fully all evaluated DMUs and identify efficient DMUs, which belong to *E*^++^ or *E*^+^ according to these rules.

For this group of seven suppliers, labeled Sup 1 through Sup 7, we gather their information about four input variables and five output variables. The results of applying the forward-stepwise approach to the stepwise DEA modeling are detailed in Table 9 and described below.

**Table 9:** Results of the forward-stepwise approach.

Step		Efficiency classification							Number of efficient DMUs
		Sup 1	Sup 2	Sup 3	Sup 4	Sup 5	Sup 6	Sup 7	
**Start**	SE_2_, SE_6_	[0.20, 0.69]	[0.29, 0.94]	[0.60, 1]	[0.29, 0.93]	[0.40, 1]	[0.31, 1]	[0.18, 0.64]	Q* = 3
	SE_2_, SE_7_	[0.47, 1]	[0.65, 1]	[0.57, 1]	[0.57, 1]	[0.51, 1]	[0.3801, 1]	[0.40, 1]	
	SE_2_, SE_8_	[0.20, 1]	[0.23, 1]	[0.27, 1]	[0.25, 1]	[0.79, 1]	[0.18, 0.93]	[0.14, 0.67]	
**Step 1** (SE_2_, SE_4_, SE_6_)		[0.38, 1]	[0.33, 1]	[0.60, 1]	[0.29, 0.99]	[0.45, 1]	[0.58, 1]	[0.27, 0.87]	*Q*_1_ = 5
**End** (SE_2_, SE_6_)		[0.20, 0.69]	[0.29, 0.94]	[0.60, 1]	[0.29, 0.93]	[0.40, 1]	[0.31, 1]	[0.18, 0.64]	

Start: Run a series of three DEA analyses by using SE_2_ as output and incorporating SE_6_, SE_7_, and SE_8_ as inputs. For each of the three analyses, we record the number of efficient DMUs separately and choose the minimal number of efficient DMUs with corresponding indicators. In this example, we can obtain the number of efficient DMUs Q* = 3 and let *C* = {SE_2_, SE_6_}.

Step 1: We now run a DEA analysis, adding the input variable SE_4_ and incorporating relative importance degrees of SE_2_ and SE_4_ as constraints. We obtain the number of efficient DMUs *Q*_1_ = 5. As *Q*_1_ > Q*, C* = {SE_2_, SE_6_}. Stop.

#### Step 2c. Select the best supplier(s) using interval DEA

According to Table 9, we obtain the final indicator system C* = {SE_2_, SE_6_}. Three efficient suppliers are indicated, which are Sup 3, Sup 5, Sup 6, and they all belong to *E*^+^. According to the rule that the smaller the difference $h_{j}^{U}-h_{j}^{L}$, the higher the ranking, we know that Sup 3 ranks first, followed by Sup 5 and Sup 6. Therefore, when companies want to choose one supplier, the best supplier is Sup 3, which should be selected as the only supplier.

### Sensitivity analysis

In order to verify the robustness of the proposed methodology, we can make a sensitivity analysis. To do this, the interval DEA models based on C* = {SE_2_, SE_6_} will be run by adding every remaining indicator respectively. The results of the proposed model after adding the indicators are shown in Table 10.

**Table 10:** Results of sensitivity analysis to indicators for the 7 suppliers.

Sup	SE_1_	SE_3_	SE_4_	SE_5_	SE_7_	SE_8_	SE_9_
Sup 1	[0.4,1]	[0.26,0.84]	[0.38,1]	[0.35,1]	[0.47,1]	[0.23,1]	[0.38,1]
Sup 2	[0.36,1]	[0.29,0.94]	[0.33,1]	[0.36,1]	[0.65,1]	[0.32,1]	[0.55,1]
Sup 3	[0.6,1]	[0.6,1]	[0.6,1]	[0.6,1]	[0.64,1]	[0.6,1]	[0.6,1]
Sup 4	[0.29,1]	[0.3,0.98]	[0.29,0.99]	[0.34,1]	[0.58,1]	[0.33,1]	[0.45,1]
Sup 5	[0.4,1]	[0.56,1]	[0.45,1]	[0.4,1]	[0.54,1]	[0.79,1]	[0.54,1]
Sup 6	[0.64,1]	[0.46,1]	[0.58,1]	[0.46,1]	[0.41,1]	[0.32,1]	[0.44,1]
Sup 7	[0.35,1]	[0.28,0.87]	[0.27,0.87]	[0.33,1]	[0.4,1]	[0.2,0.75]	[0.31,1]

First of all, according to Table 10, by adding every remaining indicator respectively, some suppliers become efficient. Based on indicator system C* = {SE_2_, SE_6_}, three efficient suppliers are indicated, which are Sup 3, Sup 5, Sup 6, and they all belong to *E*^+^. If adding SE_1_, SE_5_, SE_7_, or SE_9_, then all suppliers will be efficient in a maximal sense. However, for the efficient suppliers based on indicator system C* = {SE_2_, SE_6_}, i.e., Sup 3, Sup 5, Sup 6, they are always efficient. Secondly, according to the rule that the smaller the difference $h_{j}^{U}-h_{j}^{L}$, the higher the ranking, Sup 3 has better performance than other efficient suppliers in a wide range (adding SE_3_, SE_4_, SE_5_, SE_9_ respectively). Therefore, Sup 3 is sensitive to three indicators among seven remaining indicators, i.e., SE_1_, SE_7_, SE_8_. What’s more, when adding SE_7_ into the model, Sup 2 will be the best supplier which has the best performance in the efficiency score. That is, addition of earlier business relationship (SE_7_) has the greatest influence on the efficiency scores. To sum up, we can find that the DEA algorithm is sensitive to the number of input and output criteria in the model and excessive input or output variables will reduce the effectiveness of DEA ranking. Therefore, our proposed methodology can handle the curse of dimensionality problem efficiently.

## Conclusions

Aiming at the problem of supplier selection, this study proposes a new methodology combining fuzzy QFD and interval DEA. The proposed method comprises two phases: HOQ construction for screening indicators and evaluation of candidate suppliers, including several distinct steps within each phase. In our approach, fuzzy QFD is used to prioritize the SEs according to the CRs for products. Interval DEA is then applied to this set of prioritized criteria to screen evaluation indicators by using the forward-stepwise approach according to the relative importance degrees obtained from QFD and determine the best supplier. Through interval analysis, this study effectively solves the problem of fuzzy supplier selection.

To the best of our knowledge, our study is the first attempt to overcome the curse of dimensionality problem and screen the evaluation indicators by combining fuzzy QFD and interval DEA. On the basis of fuzzy QFD, we determine SEs and their relative importance degrees. On the basis of interval DEA, we apply the forward-stepwise approach to screen SEs according to their relative importance degrees and discrimination power. The integrated forward-stepwise approach provides a new insight for solving the curse of dimensionality in DEA.

In future research, the proposed model can be applied to real-life examples. A subsequent problem is that the use of such a model is a little complicated, especially in the phase of screening evaluation criteria. Decision makers may have their own preference for indicator selection when screening evaluation criteria. Therefore, an interactive and integrated supplier evaluation system is needed to deal with personal preference and complexity. Besides, in this study, we do not discuss that our proposed methodology is the best approach to deal with the problem of supplier selection. As there are many approaches that have been applied to supplier selection, such as AHP, ANP, TOPSIS, and so on, a comparison analysis between all these approaches is very necessary, which will be the focus of future research.
